# Proposal for a fourth indicator on vaccine uptake in the joint external evaluation tool

**DOI:** 10.2471/BLT.24.292086

**Published:** 2025-01-20

**Authors:** Lawrence R Stanberry, Tariro Makadzange, Wilmot G James, Janan J Dietrich, Peter J Hotez, Shabir A Madhi, Susan L Rosenthal, Martin Veller, Natalia Pasternak Taschner, James W Le Duc, Youngmee Jee, Anthony L Cunningham, Margie Danchin, Julie Leask

**Affiliations:** a Department of Pediatrics, Vagelos College of Physicians and Surgeons, Columbia University, 622 West 168th Street, PH-17, New York, 10032, United States of America (USA).; b Mutala Trust, Harare, Zimbabwe.; c School of Public Health, Brown University, Providence, USA.; d African Social Sciences Unit of Research and Evaluation (ASSURE), University of the Witwatersrand, Johannesburg, South Africa.; e National School of Tropical Medicine, Baylor College of Medicine, Houston, USA.; f South African Medical Research Council Vaccines and Infectious Diseases Analytics Research Unit, University of the Witwatersrand, Johannesburg, South Africa.; g Faculty of Health Sciences, University of the Witwatersrand, Johannesburg, South Africa.; h Instituto Questão de Ciência, São Paulo, Brazil.; i University of Texas Medical Branch, Galveston, USA.; j Korea Disease Control and Prevention Agency, Osong, Republic of Korea.; k Centre for Virus Research, The Westmead Institute for Medical Research, Westmead, Australia.; l Vaccine Clinical trials and Uptake Group, Murdoch Children’s Research Institute, Melbourne, Australia.; m School of Public Health, University of Sydney, Sydney, Australia.

Ensuring maximum uptake for vaccines – whether new, routine or pandemic – is essential to provide protection from known diseases and ones we may face in the future. During the coronavirus disease 2019 (COVID-19) pandemic, once vaccines were developed, lives were lost because people were unimmunized, indicating the serious challenges many countries experienced in attaining high uptake. During this time, countries also experienced the worst global decline in childhood vaccine coverage and vaccine confidence in 15 years.^1^ Between 2019 and 2021, 67 million children missed vaccinations, mostly in the World Health Organization (WHO) African and South-East Asian Regions, resulting in 9 million measles cases and more than 100 000 related deaths in 2021 alone. As such, WHO, the United Nations Children’s Fund (UNICEF) and other global partners have called for efforts to catch up on missed childhood vaccinations during the COVID-19 pandemic, restore routine immunization coverage, strengthen primary health care and build vaccine confidence.^2^

In the wake of the pandemic, global efforts are underway to better prepare for the next outbreak. As part of their 2022–2026 pandemic plan, the Coalition for Epidemic Preparedness Innovations developed the 100 days’ mission, committing to develop safe and effective vaccines within 100 days of identification of a new threat.^3^ However, if vaccines are to be beneficial routinely as well as during future pandemics, countries will need to systematically collect, synthesize and apply data on the social and behavioural drivers of vaccine uptake, alongside vaccine coverage. Identifying coverage gaps is insufficient; understanding why these gaps exist is essential. Global investment in new vaccine development is substantial. The same investment is needed to ensure that vaccines equitably reach everyone, everywhere.

## Drivers of vaccine uptake

A range of factors influence vaccine uptake. Measuring the barriers that are amenable to intervention enables governments and communities to address them in an evidence-informed way. The main drivers of low coverage are lack of service access, utilization and acceptance. Understanding the relative contribution of each driver informs targeted and cost-effective interventions. These barriers were highlighted during COVID-19 vaccine rollouts, with access problems such as vaccine shortages, long wait times and fear of accessing health services as key issues.^4^ Vaccine confidence was affected by vaccine safety and effectiveness concerns, reduced trust in government and institutions, disapproval of vaccine mandates, conspiracy beliefs and certain religious beliefs and political orientations. The presence of these factors amplified the impact of misinformation and disinformation.^5^ The ability to identify, with data, the range of influences on vaccine uptake is the most effective way to identify and prioritize strategies to increase uptake.

To standardize efforts to understand and measure the drivers of vaccination uptake, WHO and UNICEF released the *Behavioural and social drivers of vaccination: tools and practical guidance* in 2022.^6^ The guidance, developed for children and adults, includes a set of validated survey questions and qualitative interview guides that measure the modifiable factors that influence vaccination. The tools are based on four domains: how people think and feel; social processes; motivation (intention) to vaccinate; and practical issues. Hesitancy resides in the motivational domain and is defined as a state of being conflicted about or opposed to getting vaccinated.^7^ Expertise in the use of these tools is currently limited, and WHO has invested in efforts to assist countries to apply them to inform programmes.^8^

Through use of these tools and other data collection methods tailored to context, countries can systematically collect and analyse data on the main drivers of vaccine uptake from the perspective of vaccine recipients, and use these data to guide programme planning, implementation and evaluation. Progress in these efforts is reported annually through the WHO/UNICEF joint reporting form on immunization, with 103 countries reporting – including any behavioural and social drivers surveys since 2020.^9^

## The joint external evaluation tool

The joint external evaluation, developed in 2016 by WHO and UNICEF, helps countries assess their ability to prevent, detect and rapidly respond to public health risks such as infectious disease outbreaks. Such prevention, detection and response efforts are required under the *International Health Regulations* 2005 (IHR). The joint external evaluation supports country self-assessment of pandemic preparedness with 56 indicators across 19 technical areas. The technical area covering immunization evaluates a country’s vaccine delivery system including its ability to respond to new disease threats. This section includes 20 extensive technical questions grouped around three indicators: (i) vaccine coverage (measles) as part of national programmes; (ii) national vaccine access and delivery; and (iii) mass vaccination for epidemics of vaccine-preventable diseases. One of the questions covers monitoring public perception of vaccines, whether messages are tailored to different groups within vaccination campaigns and investigating barriers to uptake. 

Because measuring the drivers of vaccine uptake can improve pandemic preparedness, here we suggest integration of the behavioural and social driver indicators into the joint external evaluation. The evaluation currently lacks a unique indicator that assesses a country’s capacity to collect data on the drivers of vaccine uptake – such as why coverage is low among certain populations or geographic groups – and act on that data. In the evaluation, a separate technical area, risk communication and community engagement, poses 44 questions surrounding systems for emergencies, risk communication and community engagement capacity. This area is highly relevant to immunization, but is similarly not captured within the immunization indicators and is just one element of improving uptake. Countries need systems to build and sustain confidence in the vaccines and to ensure citizens can readily access the services delivering them.

Therefore, the important measures, strategies and capacity to rapidly improve uptake of a new pandemic vaccine are found diffusely in different sections of the joint external evaluation tool or are not sufficiently addressed. 

## New immunization uptake indicator

We suggest that stakeholders consider the adoption of a fourth immunization indicator that would evaluate countries’ capacity for measuring the drivers of routine immunization uptake and ability to use the data to inform interventions to improve uptake (Box 1). This new indicator would be directly related to the first two indicators and why they may not be met: (i) measles-containing vaccine, dose one coverage at 12 months; and (ii) national vaccine access and delivery. As such, the new fourth indicator would work to provide data to formulate responses to gaps identified in the first and second indicators through understanding the social and behavioural drivers underpinning low vaccine uptake. The indicator would also identify barriers to service delivery and other factors that affect coverage to guide programme planning and strategies to improve uptake. The promotion of the new indicator would highlight the need for countries to allocate funding to build the capacity to undertake activities to understand the social and behavioural drivers (Table 1). Two existing programmes support countries in the development of the infrastructure, strategies, trained personnel and operations needed to implement the work required by the indicator – the Global Health Security Agenda^10^ action package on immunization and the WHO/UNICEF Technet-21 programme.^11^

Box 1 **Components of a potential fourth indicator for the joint external evaluation tool**

**Drivers of vaccine uptake**

- Mechanisms in place to collect data on the behavioural and social drivers of vaccination.
- Evidence of routine data gathering and other insights from relevant stakeholders to inform programme planning.
- Evidence of programme planning to support optimization of person-centred immunization service delivery and strategies to sustain and increase confidence in vaccination.
- Evidence of implementation and evaluation of strategies to increase coverage.

**Table 1:** Example of behavioural and social drivers of vaccination priority questions and indicators pertaining to childhood vaccination

Domain	Construct	Question	Response option	Indicator
Thinking and feeling	Confidence in vaccine benefits	How important do you think vaccines are for your child’s health?	Not at all important A little important Moderately important Very important	% of parents or caregivers who say that vaccines are moderately or very important for their child’s health
Social processes	Family norms	Do you think most of your close family and friends want you to get your child vaccinated?	No Yes	% of parents or caregivers who say most of their close family and friends want their child to be vaccinated
Motivation	Intention to get child vaccinated	[COUNTRY NAME] has a schedule of recommended vaccines for children. Do you want your child to get none of these vaccines, some of these vaccines or all of these vaccines?	None Some All	% of parents or caregivers who want their child to get all of the recommended vaccines
Practical issues	Know where to get child vaccinated	Do you know where to go to get your child vaccinated?	No Yes	% of parents or caregivers who know where to get their child vaccinated
Practical issues	Affordability	How easy is it to pay for vaccination? When you think about the cost, please consider any payments to the clinic, the cost of getting there, plus the cost of taking time away from work.	Not at all easy A little easy Moderately easy Very easy	% of parents orcaregivers who say it is moderately or very easy to pay for vaccination for their child

Notes: The full set of survey questions in addition to the qualitative interview guides can be found in the full guidance.^6^ The wording of questions and indicators can be modified based on the specific population and vaccine.

The new indicator would cross-reference other relevant indicators, including risk communication and community engagement, but would be specific to immunization. For example, countries should have communication plans in place to manage adverse events following immunization, and communication expertise within designated committees. Also, efforts such as the systems to reach marginalized populations using culturally appropriate practices, currently under the joint external evaluation second immunization indicator of national vaccine access and delivery, can be brought into the new indicator. The new indicator would not directly address misinformation as this falls under the risk communication indicator. Countries would need to do deeper qualitative work to understand the role of contexts such as political orientation or religious affiliation. 

A well-organized national programme designed to enhance vaccine uptake with the goal of ensuring timely and equitable uptake of vaccines would facilitate pandemic planning and responses in keeping with the goals of the IHR (2005), and would be useful in improving uptake of existing vaccines. Such a programme would have the flexibility to adapt to new vaccines, pandemic vaccines and targeted groups. In demonstrating capacity for data collection on routine immunization, countries show they have a platform to rapidly deploy adapted questions about pandemic vaccines in the field when needed. An example is that of the WHO behavioural and social drivers tools, initially developed for child vaccination in 2019. The working group tasked with their development adapted these questions for adult COVID-19 vaccination, and the new data collection tools were used by many countries from 2021.

## Optimizing success

Optimizing vaccine uptake is a global health security concern. The proposed fourth immunization indicator would measure the drivers of vaccine uptake to inform interventions that address practical barriers and hesitancy, to increase vaccine uptake. Countries may be more likely to collect the data under the new fourth indicator if they can see how such data can be directly linked to actionable strategies. Once embedded in the joint external evaluation, clear communication with countries on its additional value and support to collect the data would be needed. Creation of a sustainable national infrastructure for countries to support improving confidence (including addressing misinformation) and uptake requires global coordination and investment of resources. This infrastructure needs to be sensitive to existing capacity in low-resource settings and optimize opportunities to engage with global efforts, such as the Global Vaccine Demand Hub^12^ capacity-building resources and additional philanthropic support. Vaccines only work if they reach those in need. Optimizing vaccine acceptance and access can help improve health and reduce mortality worldwide.
